# Comparison of opioid-free versus opioid-based total intravenous anaesthesia in elderly patients undergoing short-duration surgery: a randomized controlled trial

**DOI:** 10.1080/07853890.2025.2600751

**Published:** 2026-02-08

**Authors:** Shirong Wei, Sitong Zhou, Junwen Tu, Tong Zhi, Yungong Wang, Qihong Shen, Ming Yao, Chaobo Ni, Huadong Ni

**Affiliations:** Department of Anesthesiology and Pain Research Center, The Affiliated Hospital of Jiaxing University, Jiaxing China

**Keywords:** Opioid-free anaesthesia, Postoperative complications, Elderly, Randomized controlled trial, Total intravenous anaesthesia

## Abstract

**Introduction:**

Older adults who undergo short-duration surgery are vulnerable to opioid-related complications. It is uncertain whether an opioid-free total intravenous anaesthesia (OFA) can reduce these events. We aimed to determine whether OFA reduces the incidence of major postoperative adverse events compared with standard opioid-based total intravenous anaesthesia (OBA).

**Patients and Methods:**

This single-center randomized clinical trial was conducted in China. From May to August 2025, 400 patients aged ≥60 years undergoing elective, short-duration surgery (anticipated duration of less than 90 min) were randomized 1:1 to receive either OFA (*n* = 200) or OBA (*n* = 200). The primary outcome was a composite of postoperative hypoxemia, delirium, or nausea and vomiting (PONV) within 48 h.

**Results:**

A total of 400 randomized patients (mean [SD] age, 69.5 [7.0] years; 125 [31.3%] women). The primary composite outcome occurred in 50 patients (25.0%) in the OFA group and 87 patients (43.5%) in the OBA group (adjusted odds ratio, 0.40; 95% CI, 0.25 to 0.62; *p* < .001). Among the OFA group had a lower incidence of hypoxemia (15.0% vs 32.0%) and PONV (8.0% vs 16.0%). Intraoperative hemodynamic stability was greater in the OFA group. However, the OFA group had a higher incidence of intraoperative bradycardia (10.0% vs 3.0%; *p* = .005) and longer extubation times (mean, 9.5 vs 7.2 min; *p* < .001).

**Conclusion:**

These findings suggest that OFA is a viable alternative to opioid-based anesthesia for improving postoperative outcomes by reducing the incidence of hypoxemia and PONV in this population, while warranting careful management of its associated side effects.

**Trial registration:**

Chinese Clinical Trial Registry, ChiCTR2500102550.

## Introduction

As the global population ages, an increasing proportion of elective surgical patients are older adults [1,2]. Although clinical attention has traditionally focused on the risks associated with major, lengthy surgeries, a substantial and often under-recognized burden of morbidity also follows short-duration elective surgeries [3]. Older adults are at an increased risk of postoperative complications because of reduced physiological reserve and comorbidities [4]. Among these, opioid-related adverse events, including postoperative hypoxemia, PONV, and postoperative delirium (POD), are particularly common in this population, and can disproportionately delay recovery from procedures that would otherwise be expected to have a rapid course [5,6]. These concerns underscore the need to re-evaluate standard anesthetic practices in older patients, even for short-duration surgeries.

Conventional general anaesthesia, which typically combines inhaled anesthetics with intravenous opioids, is limited by the adverse effects of both drugs. Inhaled anesthetics may contribute to PONV and postoperative cognitive dysfunction [7,8], whereas opioids are strongly associated with respiratory depression, PONV, and POD, especially in older adults [9–11]. These concerns have led to an interest in opioid-free total intravenous anaesthesia (OFA) as a potentially feasible alternative. This approach is based on the two components: First, an opioid-free multimodal regimen using non-opioid adjuncts, such as dexmedetomidine, lidocaine, and esketamine, to provide analgesia while avoiding opioid-related harms [12]. Esketamine, a non-competitive N-methyl-D-aspartate (NMDA) receptor antagonist, is particularly because of its ability to attenuate central sensitization and provide analgesia with cardiovascular stability [13,14]. Second, the use of total intravenous anaesthesia (TIVA) avoids the risks of inhaled anesthetics [15]. By combining these two concepts, OFA has the potential to provide adequate anaesthesia while simultaneously mitigating the risks of both opioids and inhaled agents, although its clinical benefits in the older surgical populations require rigorous validation in randomized controlled trials.

Previous trials of opioid-free or opioid-based strategies have been heterogeneous in terms of patient populations, anesthetic methods, and outcomes, with few focusing specifically on short-duration procedures in the elderly [16]. High-quality randomized data are lacking to establish whether OFA can meaningfully reduce opioid-related complications while maintaining anesthetic adequacy in this population. Therefore, we conducted a single-center randomized controlled trial to compare OFA and opioid-based total intravenous anaesthesia (OBA) in older adults undergoing short-duration surgery. The primary outcome was a composite of opioid-related adverse events (postoperative hypoxemia, PONV, and POD) within 48 h postoperatively. We hypothesized that OFA would reduce the risk of the primary composite outcome compared with OBA.

## Patients and methods

### Study design

This investigator-initiated, prospective, single-center, parallel-group randomized controlled trial (RCT) was conducted at the Affiliated Hospital of Jiaxing University in China (chictr.org.cn ChiCTR2500102550; May 20, 2025). The trial protocol is available in Supplement 1. This study was approved by the Ethics Committee of the Affiliated Hospital of Jiaxing University (April 30, 2025). Written informed consent was obtained from all participating patients before inclusion in the study. We followed the Consolidated Standards of Reporting Trials (CONSORT) reporting guideline [17].

### Participants

Eligible participants were patients aged 60 years or older scheduled to undergo elective, non-major surgery with an anticipated duration of less than 90 min (e.g. urologic procedures such as lithotripsy, transurethral resection of bladder tumors [TURBT], or transurethral resection of the prostate [TURP], and vascular procedures such as great saphenous vein stripping). All patients were required to be classified as American Society of Anesthesiologists (ASA) physical status I to III, and planned for general anaesthesia with a laryngeal mask airway (LMA). Only patients who provided written informed consent were eligible for enrollment.

The exclusion criteria were as follows: contraindications to the use of LMA or a predicted difficult airway, severe cardiovascular disease (e.g. unstable angina, New York Heart Association class III-IV heart failure), severe hepatic or renal dysfunction (e.g. Child-Pugh class C, or an estimated glomerular filtration rate <30 mL/min), and uncontrolled diabetes (defined as HbA1c >9%). To avoid confounding the assessment of our primary outcome, patients with significant pre-existing cognitive impairment (Mini-Mental State Examination score <18), a history of major psychiatric disorders, or chronic opioid use or dependency were also excluded. Further exclusion criteria included known allergies to any of the study medications, participation in another interventional trial within 30 days, or refusal to provide consent.

### Blinding and randomization

Participants were randomly assigned in a 1:1 ratio to the OFA or OBA groups. A statistician not involved in the study generated the allocation sequence using SPSS software (version 26.0) with a block size of 4 or 6. To ensure allocation concealment, the assignments were placed in sequentially numbered, sealed, opaque envelopes. On the day of surgery, after the participant provided consent, an independent research assistant selected the next appropriate envelope. An unblinded anesthesiologist then opened it to reveal the treatment allocation. All other individuals, including the participants, outcome assessors, and data analysts, were blinded to the group assignment throughout the trial. To minimize potential bias, the unblinded anesthesiologists were not involved in any postoperative data collection or outcome assessments.

### Outcome

The primary outcome was a composite of major opioid-related adverse events occurring within the first 48 h after surgery. The components of the primary outcome were [1]: postoperative hypoxemia, defined as an Spo_2_ level of less than 95% with a need for oxygen supplementation [2,18]; POD, assessed as positive using the 3-Minute Disorientation Assessment Method (3D-CAM) (eAppendix 5 in **Supplement 2**) [19]; and [3] PONV, defined as any episode of nausea, retching, or vomiting.

The secondary outcomes were measures of intraoperative hemodynamic stability and postoperative recovery. Hemodynamic stability was assessed using the area under the curve for mean arterial pressure (MAP AUC) relative to baseline during the first 15 min of anaesthesia induction and the requirement for intraoperative vasopressors. Postoperative recovery outcomes included the time to LMA removal, the length of stay in the post-anaesthesia care unit (PACU), and the length of hospital stay, and the severity of PONV, which was assessed using a 4-point ordinal scale. The grades were defined as follows: 0 (none), no nausea or vomiting reported; 1 (mild), subjective nausea without vomiting and not requiring rescue antiemetic therapy; 2 (moderate), vomiting of 1 to 2 episodes or persistent nausea requiring rescue antiemetic therapy; and 3 (severe), vomiting of more than 2 episodes or nausea and vomiting that were refractory to rescue antiemetic therapy [20]. Postoperative pain was assessed using the Numerical Rating Scale (NRS) at rest and exercise in the PACU. Safety outcomes included the incidence of injection pain during induction, as well as other adverse events such as significant bradycardia or tachycardia, dizziness, headache, and emergence agitation.

To ensure data quality and minimize missing data, a trained research coordinator, who was blinded to the treatment allocation and was not involved in patient care, prospectively collected all outcome data using a standardized electronic case report form from randomization until hospital discharge.

### Intervention

All patients followed standard fasting guidelines (no solid food for 6 h and no clear liquids for 2 h preoperatively). Upon arrival in the operating room, standard monitoring was performed, including electrocardiography, pulse oximetry, and non-invasive blood pressure. Analgesic depth was specifically monitored using the Surgical Pleth Index (SPI).

In OFA group, induction was performed with intravenous lidocaine (1 mg·kg^−1^), esketamine (0.2–0.4 mg·kg^−1^), and propofol (1.5–2.0 mg·kg^−1^). In OBA group, induction was performed with intravenous sufentanil (0.2–0.4 μg·kg^−1^) and propofol (1.5–2.0 mg·kg^−1^). For both groups, rocuronium (0.6–1.0 mg·kg^−1^) was used to facilitate LMA insertion.

Following LMA placement, mechanical ventilation was initiated with tidal volumes set at 6–8 mL/kg of ideal body weight and the respiratory rate was adjusted to maintain an end-tidal carbon dioxide (EtCO_2_) between 35 and 45 mmHg. Anaesthesia was maintained with a continuous infusion of propofol (2–10 mg·kg^−1^·h^−1^) in both groups. In the OFA group, analgesia was maintained with continuous infusions of esketamine (0.5 mg·kg^−1^·h^−1^) and dexmedetomidine (0.3–1.0 μg·kg^−1^·h^−1^). In the OBA group, analgesia was maintained with a continuous infusion of remifentanil (0.1–0.2 μg·kg^−1^·min^−1^). Propofol infusion was adjusted based on clinical signs, while the primary analgesic infusions were adjusted to maintain an SPI value between 20 and 50. Intraoperative hypotension (defined as MAP < 65 mmHg or a decrease >20% from baseline for more than 1 min) was treated with phenylephrine boluses. Bradycardia (heart rate < 50 beats/min) was treated with atropine.

At the end of surgery, residual neuromuscular blockade was antagonized with intravenous sugammadex, with the dose determined by train-of-four (TOF) monitoring. The LMA was removed from the operating room once the standard criteria for emergence were met, including regular spontaneous respiration, adequate tidal volumes, and response to verbal commands. Postoperative care in the PACU was standardized for both groups. Pain was assessed using the NRS, and patients with an NRS score > 3 received intravenous non-steroidal anti-inflammatory drug (NSAID). Ondansetron was used as a rescue medication for postoperative nausea and vomiting. Patients were subsequently transferred from the PACU to the ward once they met standard discharge criteria.

### Sample size

The sample size for this trial was determined based on the primary composite outcome. Based on the published literature, the incidence of opioid-related adverse events in this patient population is approximately 20% for postoperative hypoxemia, 15% for PONV, and 5% for POD [21–23]. Accordingly, we estimated the incidence of the primary composite outcome to be approximately 40% in the OBA group. We considered a relative risk reduction of 40% (i.e. a reduction in the primary outcome incidence from 40% to 24%) to be a clinically meaningful treatment effect. A sample size of 130 patients per group was required to detect this difference with 80% power at a two-sided alpha level of 0.05. We increased the target sample size to 400 participants (200 per group) to account for potential dropouts and ensure adequate power for prespecified subgroup analyses.

### Statistical analysis

All analyses will be conducted according to the intention-to-treat (ITT) principle, in which all randomized participants will be analyzed in the group to which they were originally assigned, regardless of protocol adherence or the treatment ultimately received. Baseline demographic and clinical characteristics will be summarized to assess the balance achieved between the two groups by randomization. Continuous variables were presented as means with standard deviations (SDs) or medians with interquartile ranges (IQRs), while categorical variables were presented as frequencies and percentages. Between-group comparisons were performed using the independent samples t-test, the Mann-Whitney U test, or the χ^2^ test (or Fisher’s exact test, as appropriate).

The primary outcome was a composite binary endpoint, defined as the occurrence of at least 1 prespecified opioid-related adverse event within 48 h after surgery. The primary hypothesis was tested using a multivariable logistic regression model to estimate the adjusted odds ratio (aOR) and 95% confidence interval (CI) for the OFA group compared with the OBA group. The model was adjusted for six baseline covariates: age, sex, body-mass index (BMI), ASA physical status, age adjusted Charlson Comorbidity Index (aCCI), and educational level. To account for potential nonlinear relationships with the outcome, age and BMI were modeled using restricted cubic splines. To quantify the clinical effect size, a supplementary analysis was conducted. We used a modified Poisson regression model with a robust error variance to directly estimate the adjusted relative risk (aRR) after adjusting for the same covariates. The absolute risk reduction (ARR) and the number needed to treat (NNT), with their corresponding 95% CIs, were then calculated from the aRR. Missing data on the primary outcome were handled using multiple imputation.

A comprehensive assessment of efficacy was conducted by analyzing a series of secondary outcomes. For the key hemodynamic outcome—the area under the curve for the MAP AUC during the first 15 min after anaesthesia induction—an analysis of covariance (ANCOVA) model was used, with the baseline MAP value included as a continuous covariate to improve statistical precision. For all other secondary outcomes, continuous variables were compared using the independent samples t-test or the Mann-Whitney U test; ordinal outcomes were analyzed with the Mann-Whitney U test, and binary outcomes, including the individual components of the primary composite endpoint, were compared using the χ^2^ test or Fisher’s exact test. Safety outcomes, including the incidence of adverse events, such as bradycardia, tachycardia, headache, dizziness, and emergence agitation, were also compared between the groups using the χ^2^ test or Fisher’s exact test.

Two exploratory analyses were conducted. To identify potential predictors of the primary composite endpoint, we performed separate univariable logistic regression analyses for each baseline and clinical characteristic, reporting unadjusted odds ratios and 95% CIs. Second, to assess the dose–response relationship between cumulative propofol exposure and time to extubation, we fitted a linear regression model with RCS to accommodate potential nonlinearity.

We performed a series of subgroup analyses of the primary outcome to explore the potential heterogeneity of the treatment effect (i.e. effect modification) across patient characteristics. The primary subgroup analysis was based on the type of surgery (urological vs non-urological). We also explored subgroup effects based on age (<75 vs ≥75 years) and the aCCI (<5 vs ≥5). These analyses were performed by including an interaction term between the treatment assignment and the subgrouping variable in the primary univariable logistic regression model. We primarily focused on the P value for the interaction to assess for statistically significant effect modification and presented the aORs and 95% CIs for each subgroup in a forest plot. To test the robustness of the primary findings under different analytical assumptions, we conducted a series of sensitivity analyses. First, to assess the influence of protocol deviations related to surgical duration, we repeated the primary analysis on a subgroup of patients whose surgical duration was strictly within the 90-minute protocol definition. Second, to evaluate the overall impact of covariate adjustment, we fitted a univariable model containing only the treatment assignment and compared its effect estimate (crude odds ratio) with that of our primary multivariable model (aOR).

## Results

### Patients

**Figure 1.**
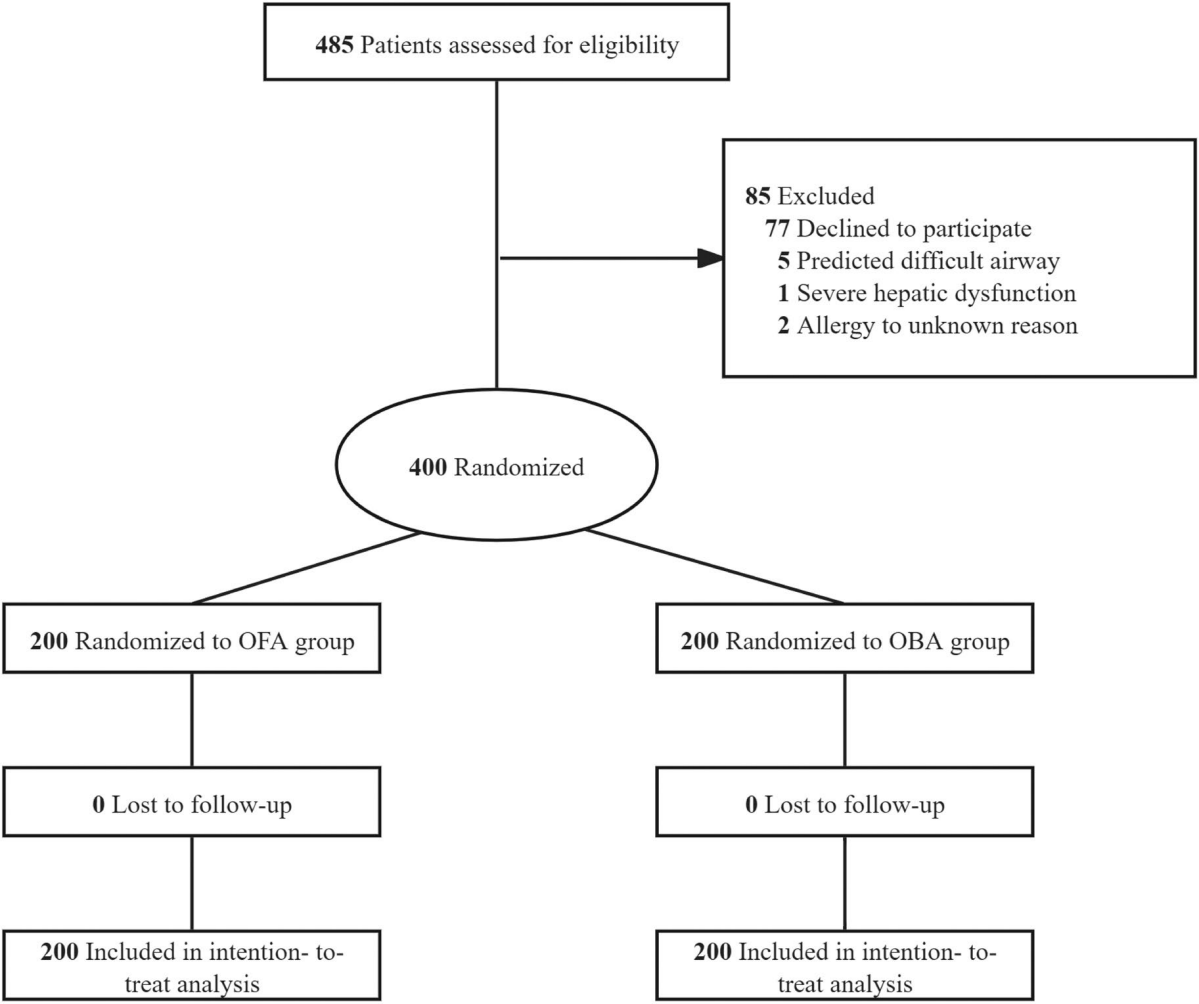
••••.

From May to August 2025, a total of 485 patients were screened for eligibility in this single-center trial. Of these, 85 patients were excluded, primarily because they declined to participate (*n* = 77). The 400 eligible patients were randomized in a 1:1 ratio to receive either OFA (*n* = 200) or OBA (*n* = 200). All randomized patients completed the study and were included in the ITT analysis, and no participants were lost to follow-up. Seven patients had a surgery duration that exceeded the 90-minute protocol definition (6 in the OFA group and 1 in the OBA group). The participant flow chart is shown in Figure 1.

### Baseline and clinical characteristics

A total of 400 randomized patients were included in the intention-to-treat analysis (OFA, *n* = 200; OBA, *n* = 200). Baseline demographics were generally similar between two groups (Table 1). The duration of surgery was similar between the two groups (median [IQR], 32 [17] min for the OBA group vs 33 [22] min for the OFA group). As per the study protocol, patients in the OBA group received sufentanil and remifentanil, while patients in the OFA group received esketamine, dexmedetomidine, and lidocaine. Propofol administration was significantly higher in the OFA group than in the OBA group (median [IQR], 404 [160] mg vs 351 [118] mg; *p* < .001). The percentage of time that the SPI was maintained within the target range was high and comparable in both groups.

**Table 1.** Baseline and peri-operative data of patients^a^.

	No. (%)	
Variable	OFA (*n* = 200)	OBA (*n* = 200)
Age,y, Mean ± SD,y	70 ± 7	69 ± 7
BMI, Mean ± SD^b^	23.5 ± 3.6	24.3 ± 3.0
APFEl score, Mean ± SD^c^	1.18 ± 0.92	1.22 ± 0.98
ASA physical status		
II	181 (90.5%)	179 (89.5%)
III	19 (9.5%)	21(10.5%)
Type of surgery		
urological surgery	149 (74.5%)	148 (74.0%)
Vascular surgery	51 (25.5%)	52 (26.0%)
Gender		
Male	133 (66.5%)	142(71.0%)
Female	67 (33.5%)	58 (29.0%)
Educational level,y		
≤6	116 (58.0%)	118 (59.0%)
6–9	67 (33.5%)	65 (32.5%)
>9	17 (8.5%)	17 (8.5%)
Drinking alcohol	39 (19.5%)	41 (20.5%)
smoking	53 (26.5%)	50(25.0%)
Comorbidities		
Hypertension	102 (51.0%)	109 (54.5%)
Diabetes	34 (17.0%)	31 (15.5%)
Atrial fibrillation	7 (3.5%)	8 (4.0%)
Coronary heart disease	12 (6.0%)	6 (3.0%)
Chronic Obstructive Pulmonary Disease	6 (3.0%)	8 (4.0%)
Prior stroke	9 (4.5%)	14 (7.0%)
Medication on admission		
β-Adrenergic receptor blocker	6 (3.0%)	7 (3.5%)
Calcium Channel Blockers	55 (27.5%)	50 (25.0%)
Lipid-lowering drug	15 (7.5%)	18 (9.0%)
Antiplatelet	27 (13.5%)	24 (12.0%)
ACEIs/ARBs	54 (27.0%)	47 (23.5%)
Diuretics	11 (5.5%)	14 (7.0%)
Age adjusted Charlson comorbidity index		
< 5	139 (69.5%)	148 (74.0%)
≥ 5	61 (30.5%)	52 (26.0%)
History of nausea and vomiting	14 (7.0%)	21 (10.5%)
Duration of surgery, median (IQR), min^d^	33 ± 22	32 ± 17
Dose of propofol, median (IQR), mg	404 ± 160	351 ± 118
Dose of esketamin, median (IQR), mg	31 ± 14	NA
Dose of dexmethomidine, median (IQR), μg	18 ± 12	NA
Dose of lidocaine, median (IQR), μg	63 ± 10	NA
Dose of sufentanil, median (IQR), mg	NA	14.4 ± 3.3
Dose of remifentanil, median (IQR), mg	NA	0.28 ± 0.18
% of Time with SPI value 20–50, median (IQR), %^e^	89.23 ± 2.70	90.83 ± 2.83

Abbreviations: ACEIs, angiotensin-converting enzyme inhibitors; APFEL, Apfel simplified risk score for postoperative nausea and vomiting; ARBs, angiotensin receptor blockers; ASA, American Society of Anesthesiologists; BMI, body mass index; OBA, opioid-based anaesthesia; OFA, opioid-free anaesthesia; SD, standard deviation.

^a^Values are reported as No. (%) unless otherwise indicated.

^b^The body-mass index (BMI) is the weight in kilograms divided by the square of the height in meters.

^c^APFEL score (range, 0–4), with higher scores indicating greater risk of postoperative nausea and vomiting; 1 point each for female sex, non-smoking status, history of nausea/vomiting or motion sickness, and use of postoperative opioids.

^d^Duration of surgery was defined as the time from skin incision to skin closure.

### Primary outcome

The primary composite outcome occurred in 50 of 200 patients (25.0%) in the OFA group and in 87 of 200 patients (43.5%) in the OBA group (Table 2). After adjusting for prespecified baseline covariates, the use of OFA was associated with significantly lower odds of the primary outcome than OBA (adjusted odds ratio, 0.40; 95% CI, 0.25 to 0.62; *p* < .001). This corresponded to an NNT of 5 (95% CI, 3–8) to prevent one additional primary outcome event.

**Table 2.** Primary outcome and its components.

Variable	No. (%)OFA (*n* = 200)	OBA(*n* = 200)	Odds Ratio (95% CI)	*p* value^a^	Adjusted Odds Ratio (95% CI)^b^	Adjusted *p* value	NNT^c^
Primary composite outcome	50(25%)	87 (43.5%)	0.43 (0.28–0.66)	<0.001	0.40 (0.25–0.62)	<0.001	5 (3–8)
Components of the primary outcome^,^							
Postoperative^d^ hypoxemia	30 (15.0%)	64 (32.0%)	0.37 (0.23–0.61)	<0.001	NA	NA	6 (4–12)
POD^e^	5 (2.5%)	5 (2.5%)	1.00 (0.28–3.51)	>0.99	NA	NA	
PONV^f^	16 (8.0%)	32 (16.0%)	0.46 (0.24–0.86)	0.014	NA	NA	13 (7–59)

Values are presented as numbers (proportions). Abbreviations: CI, confidence interval; POD; postoperative delirium; PONV: Postoperative nausea and vomiting.

^a^All tests were 2-sided. P value of less than .05 was considered significant.

^b^The adjusted odds ratio and its 95% CI were calculated using a multivariable logistic regression model. The model was adjusted for the following prespecified baseline covariates: age, gender, body mass index, ASA physical status, aCCI, educational level, age and body mass index.

^c^Derived from absolute risk difference and given only for incidence of Primary composite outcome, Postoperative hypoxemia and PONV.

^d^Postoperative hypoxemia was defined as a pulse oximetry saturation <95% requiring supplemental oxygen.

^e^POD was assessed using the 3-Minute Disorientation Assessment Method (3D-CAM).

^f^PONV included any episode of nausea (an urge to vomit), retching, or vomiting.

In prespecified analyses to assess the assumptions of our primary model, we evaluated the relationship between continuous baseline covariates and the primary outcome using restricted cubic splines. We found no evidence of a significant association with age (P for overall association = .338; P for nonlinearity = .512). For BMI, we observed a borderline significant nonlinear (J-shaped) relationship (P for nonlinearity = .051). Detailed results and graphical representations are provided in the Supplement (eFigure 1 and eFigure 2 in Supplement 2).

Among the components of the primary outcome, the incidence of postoperative hypoxemia was significantly lower in the OFA group than in the OBA group (15.0% vs 32.0%; crude OR, 0.37; 95% CI, 0.23 to 0.61; *p* < .001), with an NNT of 6 (95% CI, 4 to 12). The incidence of PONV was also significantly lower in the OFA group (8.0% vs 16.0%; crude OR, 0.46; 95% CI, 0.24 to 0.86; *p* = .014), with an NNT of 13 (95% CI, 7 to 59). There was no significant difference in the incidence of POD between two groups (2.5% vs 2.5%; *p* > .99).

### Secondary and safety outcome

**Figure 2.**
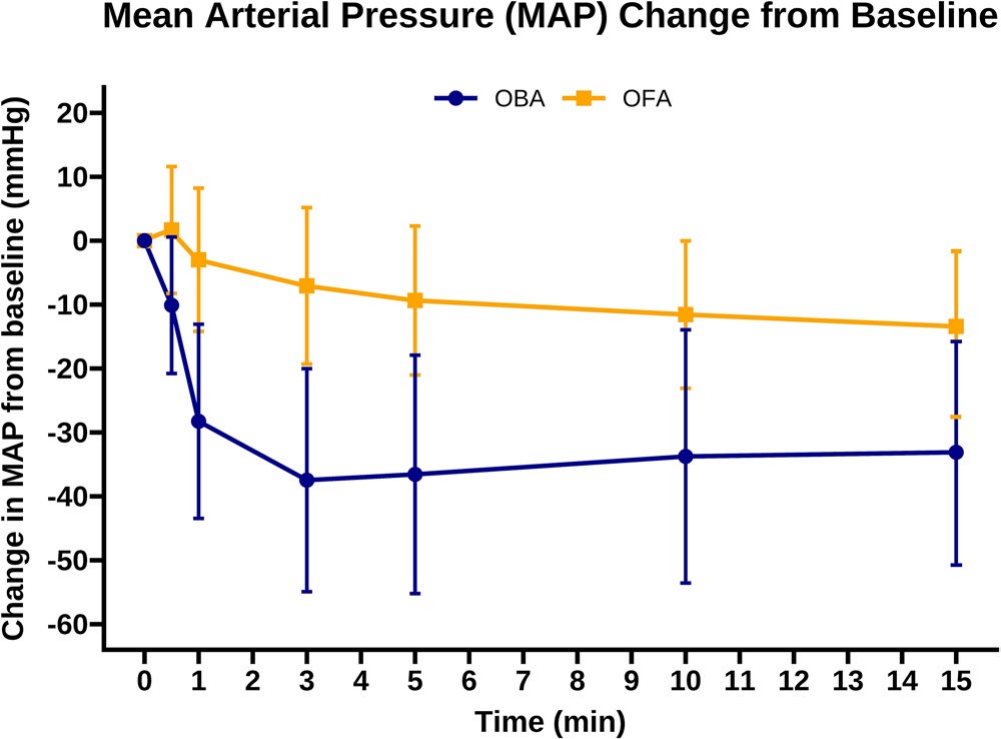
Change in Mean Arterial Pressure (MAP) From Baseline After Anesthesia Induction. Data points represent the mean change in MAP from the preoperative baseline value over the first 15 minutes of anesthesia. Error bars indicate the 95% confidence intervals. OFA, opioid-free anesthesia; OBA, opioid-based anesthesia.

The secondary and safety outcomes are shown in Table 3. Patients in the OFA group demonstrated significantly greater hemodynamic stability compared with the OBA group. Changes in MAP after induction are shown in Figure 2. The mean AUC of the difference in mean arterial pressure from baseline was smaller in the OFA group (mean, −179.0 vs −466.2 mmHg·s; *p* < .001), and the requirement for intraoperative vasopressors was significantly lower (10.0% vs 35.0%; *p* < .001). Extubation time was longer in the OFA group (mean, 9.5 vs 7.2 min; *p*<.001). The PACU length of stay and postoperative pain scores did not differ between two groups. The severity of postoperative nausea and vomiting was lower in the OFA group (*p* < .001).

**Table 3.** Secondary and safety outcomes.

	No. (%)		
Variable^a^	OFA (*n* = 200)	OBA(*n* = 200)	*p* value^b^
AUC of MAP difference from baseline, mmHg · s^c^	−179.04 (−201.20 to −156.88)	−466.17 (−488.33 to −444.01)	<0.001
Requirement for intraoperative vasopressors^d^	20 (10.0%)	70 (36.0%)	<0.001
Degree of nausea and vomiting^e^			<0.001
0	184 (92.0%)	168 (84.0%)	
1	10 (5.0%)	17 (8.5%)	
2	3 (1.5%)	12 (6.0%)	
3	3 (1.5%)	3 (1.5%)	
Length of PACU stay.min	38.4 (15.05)	37.8 (15.81)	0.73
Extubation duration min	9.5 (2.15)	7.2 (2.09)	<0.001
The NRS score at rest^f^	0.6 (0.68)	0.6 (0.79)	0.595
The NRS score at exercise	1.7 (1.37)	1.9 (1.36)	0.269
Safety Outcomes^g^			
Emergence agitation	8 (4.0%)	11 (5.5%)	0.48
Headache and dizziness	18 (9.0%)	9 (4.5%)	0.073
Injection pain	2 (1.0%)	24 (12.0%)	<0.001
Bradyarrhythmia	20 (10.0%)	6 (3.0%)	0.005
Tachycardia	5 (2.5%)	8 (4.0%)	0.40

Abbreviations: AUC, area under the curve; MAP, mean arterial pressure; NRS, numerical rating scale; PACU, post-anaesthesia care unit.

^a^Data are presented as mean (SD), median [IQR], or n (%).

^b^Between-group comparisons were performed using independent-samples t tests or Wilcoxon rank-sum tests for continuous variables and χ^2^ tests or Fisher exact tests for categorical variables, except for the ANCOVA-derived P value for MAP AUC.

^c^The AUC of the MAP difference from baseline was calculated for the first 15 min after anaesthesia induction. A more negative value indicates greater hemodynamic instability. The comparison was performed using an Analysis of Covariance (ANCOVA) model, adjusting for the baseline MAP value.

^d^Requirement for intraoperative vasopressors was defined as the need for norepinephrine bolus (5 μg) administration if mean arterial pressure (MAP) decreased by more than 20% from baseline or fell below 65 mmHg for at least 1 min.

^e^Nausea and vomiting were graded on a 4-point ordinal scale (0 = none, 1 = mild, 2 = moderate, 3 = severe).

^f^NRS, numerical rating scale (0–10, higher scores indicating greater pain).

^g^Emergence agitation was assessed during recovery. Headache/dizziness were patient-reported in the PACU. Injection pain, bradyarrhythmia, and tachycardia were defined as intraoperative adverse events documented by the anesthesiologist.

For safety outcomes, the incidence of bradyarrhythmia was significantly higher in the OFA group (10.0% vs 3.0%; *p* = .005), while injection pain was reported significantly less often (1.0% vs 12.0%; *p* < .001). There were no significant differences in the incidence of emergence agitation, headache and dizziness, or tachycardia between the groups.

### Subgroup analysis

eFigure 4 in Supplement 2 shows the results of the subgroup analyses of the effect of OFA on the primary composite outcome. No significant interactions were observed across the prespecified subgroups including type of surgery (P for interaction=.93), age (P for interaction=.15), and aCCI (P for interaction=.91). These findings indicate that the beneficial effect of OFA was consistent across subgroups, despite the numerical variation in the adjusted odds ratios. The protective effect of OFA remained statistically significant and clinically relevant in each subgroup, including in patients undergoing urological or vascular surgery and those younger or older than 75 years.

### Exploratory analysis

As prespecified, we conducted exploratory analyses to examine potential risk factors for the primary outcome and to evaluate the dose-response relationship between propofol exposure and extubation time (eAppendix 2 in Supplement 2). Univariable analyses indicated that female sex, a history of nausea or vomiting, and higher APFEL scores were associated with increased odds of the composite outcome, whereas age, BMI, hypertension, and smoking were not significantly associated (eTable 2 in Supplement 2). Restricted cubic spline analysis suggested a non-linear association between cumulative propofol dose and extubation time, with higher doses progressively associated with longer extubation times (eFigure 3 in Supplement 2). These findings are exploratory and should be interpreted with caution.

### Sensitivity analysis

The primary finding was robust across a series of sensitivity analyses. The treatment effect of OFA was consistent when the analysis was restricted to patients who met the protocol-defined surgical duration, as well as when comparing models with different covariate adjustments, and under best-case and worst-case scenarios for the 3 participants with missing data. The detailed results of all sensitivity analyses are provided in the Supplement (eTable 3–5 in Supplement 2).

## Discussion

In this randomized controlled trial involving older adults undergoing short-duration surgery, OFA significantly reduced the incidence of a composite of major postoperative adverse events compared to a standard OBA group. This benefit was primarily associated with a lower incidence of postoperative hypoxemia and PONV and was accompanied by greater intraoperative hemodynamic stability. These results suggest that OFA is a considerable alternative to OBA for enhancing perioperative safety in this patient population.

The observed differences in these outcomes have plausible mechanistic explanations. A key finding of our study was the difference in postoperative hypoxemia. The higher incidence in the OBA group (32.0%) was likely caused by the residual respiratory depressant effect of the induction opioid, sufentanil [24], given the short median surgical duration. Conversely, while the incidence was significantly lower in the OFA group (15.0%), its occurrence highlights that postoperative hypoxemia in the elderly has multiple causes. These can include the residual effects of propofol, atelectasis, or the patient’s pre-existing comorbidities. The reduction in PONV is consistent with the previous study [25]. Furthermore, the superior hemodynamic profile observed in the OFA group is likely due to the sympathomimetic properties of esketamine, which counteracts propofol-induced vasodilation. In contrast, the higher vasopressor requirement and incidence of hypotension in the OBA group can be explained by the vasodilatory effect of propofol and the sympatholytic properties of opioid. Our trial therefore provides specific evidence for shorter procedures in older adults, a patient group not extensively studied in previous OFA trials, which have often focused on major surgery [26–30]. Postoperative pain scores were equivalent between the two groups, suggesting that the OFA regimen did not offer a superior analgesic advantage over OBA.

The differing safety outcomes between the OFA and OBA groups warrant a balanced consideration. The higher incidence of bradycardia is a known pharmacodynamic effect of dexmedetomidine, a core component of our OFA intervention [31,32]. Similarly, the slightly longer extubation time may be explained by the longer context-sensitive half-time of dexmedetomidine or by the higher cumulative dose of propofol required in the OFA group. Importantly, this minor delay in extubation did not result in a longer PACU stay, indicating they were manageable in the clinical setting. Additionally, in our study, the incidence of headache and dizziness in the OFA group was double that of the OBA group, this difference was not statistically significant and also did not result in a longer PACU stay. This may be a plausible pharmacodynamic effect of esketamine [33,34].

Our findings are consistent with a recent network meta-analysis [35], which assessed the effectiveness and safety of OFA regimens. In line with their primary conclusion, our study also found no superior analgesic effect for OFA compared to OBA. However, our trial contributes direct evidence for the safety benefits of OFA by confirming a reduction in PONV—a finding also reported in the meta-analysis—and, importantly, by demonstrating a significant reduction in postoperative hypoxemia in our specific patient population. These results therefore suggest that the clinical value of OFA in elderly patients undergoing short-duration surgery is primarily related to improved perioperative safety, rather than superior analgesic efficacy.

Our study had several limitations. First, as a single-center trial, our findings may not be completely generalizable to other institutions with different clinical practices or patient populations. Secondly, while the outcome assessors were blinded, the attending anesthesiologists were not, which could produce performance bias. We sought to mitigate this by standardizing the anesthetic protocol and separating the roles of intervention delivery from the outcome assessment. Thirdly, our composite primary outcome was driven primarily by differences in hypoxemia and PONV; the study was not powered to detect a difference in the less frequent outcome of POD. Fourth, although the prevalence of mild-to-moderate COPD was balanced between groups, its inclusion could be a potential confounder for postoperative hypoxemia.

## Conclusions

In conclusion, our study demonstrates that for older patients undergoing short-duration surgery, a dexmedetomidine-esketamine-based OFA regimen is a considerable alternative to OBA. It was associated with a lower incidence of postoperative hypoxemia and PONV and provided greater hemodynamic stability, with equivalent postoperative analgesia. These benefits come with the known side effects of dexmedetomidine, including a higher incidence of bradycardia and a modest delay in emergence. These findings support the use of OFA as an alternative anesthetic choice, particularly for patients in whom minimizing opioid-related complications is a key objective.

## Supplementary Material

Supplement 1 Protocol.docx

consort flow chart.pdf

CONSORT checklist.docx

## Data Availability

The data that support the findings of this study are not openly available. Deidentified individual participant data may be made available to qualified researchers for non-commercial purposes upon reasonable request to the corresponding author, pending the submission of a research proposal and execution of a data sharing agreement.

## References

[1] Naik H, Murray TM, Khan M, et al. Population-based trends in complexity of hospital inpatients. JAMA Intern Med. 2024;184(2):183–192. doi:10.1001/jamainternmed.2023.7410. 38190179 PMC10775081

[2] Leigard E, Hertzberg D, Konrad D, et al. Increasing perioperative age and comorbidity: a 16-year cohort study at two university hospital sites in sweden. Int J Surg. 2024;110(7):4124–4131. doi:10.1097/js9.0000000000001326. 38498387 PMC11254224

[3] Seib CD, Rochefort H, Chomsky-Higgins K, et al. Association of patient frailty with increased morbidity after common ambulatory general surgery operations. JAMA Surg. 2018;153(2):160–168. doi:10.1001/jamasurg.2017.4007. 29049457 PMC5838594

[4] Khanna AK, Motamedi V, Bouldin B, et al. Automated electronic frailty index-identified frailty status and associated postsurgical adverse events. JAMA Netw Open. 2023;6(11):e2341915. doi:10.1001/jamanetworkopen.2023.41915. 37930697 PMC10628731

[5] Barreveld AM, McCarthy RJ, Elkassabany N, et al. Opioid stewardship program and postoperative adverse events: a difference-in-differences cohort study. Anesthesiology. 2020;132(6):1558–1568. doi:10.1097/aln.0000000000003238. 32167983

[6] Levy N, Quinlan J, El-Boghdadly K, et al. An international multidisciplinary consensus statement on the prevention of opioid-related harm in adult surgical patients. Anaesthesia. 2021;76(4):520–536. doi:10.1111/anae.15262. 33027841

[7] Ahn H, Chae YJ, Kang S, et al. Postoperative nausea and vomiting and recovery of heart rate variability following general anesthesia with propofol or sevoflurane: a randomized, double-blind preliminary study. Front Med (Lausanne). 2025;12:1575865. doi:10.3389/fmed.2025.1575865. 40370731 PMC12075522

[8] Aykut A, Salman N, Demir ZA, et al. Comparison of propofol and sevoflurane anaesthesia in terms of postoperative nausea-vomiting complication in cardiac surgery patients undergoing enhanced recovery after surgery protocol: a prospective randomized study. Turk J Anaesthesiol Reanim. 2024;52(3):113–121. doi:10.4274/tjar.2024.241622. 38994778 PMC11590698

[9] Algera MH, Kamp J, van der Schrier R, et al. Opioid-induced respiratory depression in humans: a review of pharmacokinetic-pharmacodynamic modelling of reversal. Br J Anaesth. 2019;122(6):e168–e79. doi:10.1016/j.bja.2018.12.023. 30915997

[10] Baldo BA. Toxicities of opioid analgesics: respiratory depression, histamine release, hemodynamic changes, hypersensitivity, serotonin toxicity. Arch Toxicol. 2021;95(8):2627–2642. doi:10.1007/s00204-021-03068-2. 33974096

[11] Huang S, Zhang L, Tian Y. Opioids worsen postoperative sleep: a narrative review. Anesthesiol Perioper Sci. 2025;3(3):32. doi:10.1007/s44254-025-00114-5.

[12] Carron M, Tamburini E, Linassi F, et al. Efficacy of nonopioid analgesics and adjuvants in multimodal analgesia for reducing postoperative opioid consumption and complications in obesity: a systematic review and network meta-analysis. Br J Anaesth. 2024;133(6):1234–1249. doi:10.1016/j.bja.2024.08.009. 39366846

[13] Ferraro MC, Cashin AG, Visser EJ, et al. Ketamine and other nmda receptor antagonists for chronic pain. Cochrane Database Syst Rev. 2025;8(8):Cd015373. doi:10.1002/14651858.CD015373.pub2. 40819842 PMC12358209

[14] Levinstein MR, Budinich RC, Bonaventura J, et al. Redefining ketamine pharmacology for antidepressant action: synergistic nmda and opioid receptor interactions? Am J Psychiatry. 2025;182(3):247–258. doi:10.1176/appi.ajp.20240378. 39810555 PMC11872000

[15] Miller D, Lewis SR, Pritchard MW, et al. Intravenous versus inhalational maintenance of anaesthesia for postoperative cognitive outcomes in elderly people undergoing non-cardiac surgery. Cochrane Database Syst Rev. 2018;8(8):Cd012317. doi:10.1002/14651858.CD012317.pub2. 30129968 PMC6513211

[16] Frauenknecht J, Kirkham KR, Jacot-Guillarmod A, et al. Analgesic impact of intra-operative opioids vs. Opioid-free anaesthesia: a systematic review and meta-analysis. Anaesthesia. 2019;74(5):651–662. doi:10.1111/anae.14582. 30802933

[17] Hopewell S, Chan AW, Collins GS, et al. Consort 2025 statement: updated guideline for reporting randomized trials. JAMA. 2025;333(22):1998–2005. doi:10.1001/jama.2025.4347. 40228499

[18] Abbott TEF, Fowler AJ, Pelosi P, et al. A systematic review and consensus definitions for standardised end-points in perioperative medicine: pulmonary complications. Br J Anaesth. 2018;120(5):1066–1079. doi:10.1016/j.bja.2018.02.007. 29661384

[19] Oberhaus J, Wang W, Mickle AM, et al. Evaluation of the 3-minute diagnostic confusion assessment method for identification of postoperative delirium in older patients. JAMA Netw Open. 2021;4(12):e2137267. doi:10.1001/jamanetworkopen.2021.37267. 34902038 PMC8669542

[20] Myles PS, Wengritzky R. Simplified postoperative nausea and vomiting impact scale for audit and post-discharge review. Br J Anaesth. 2012;108(3):423–429. doi:10.1093/bja/aer505. 22290456

[21] Zhang Z, Li C, Xu L, et al. Effect of opioid-free anesthesia on postoperative nausea and vomiting after gynecological surgery: a systematic review and meta-analysis. Front Pharmacol. 2023;14:1330250. doi:10.3389/fphar.2023.1330250. 38239201 PMC10794765

[22] Wang D, Long YQ, Sun Y, et al. Opioid-free total intravenous anesthesia for thyroid and parathyroid surgery: protocol for a randomized, double-blind, controlled trial. Front Med (Lausanne). 2022;9:939098. doi:10.3389/fmed.2022.939098. 36111120 PMC9468489

[23] da Silveira CAB, Rasador ACD, Medeiros HJS, et al. Opioid-free anesthesia for minimally invasive abdominal surgery: a systematic review, meta-analysis, and trial sequential analysis. Can J Anaesth. 2024;71(11):1466–1485. doi:10.1007/s12630-024-02831-0. 39500840

[24] Doufas AG, Laporta ML, Driver CN, et al. Incidence of postoperative opioid-induced respiratory depression episodes in patients on room air or supplemental oxygen: a post-hoc analysis of the prodigy trial. BMC Anesthesiol. 2023;23(1):332. doi:10.1186/s12871-023-02291-x. 37794334 PMC10548743

[25] Rauseo M, Mirabella L, Carrideo AA, et al. Opioid-sparing anesthesia in cardiac surgery: a meta-analysis. J Cardiothorac Vasc Anesth. 2025;39(11):3140–3153. doi:10.1053/j.jvca.2025.06.040. 40685295

[26] Wang D, Sun Y, Zhu YJ, et al. Comparison of opioid-free and opioid-inclusive propofol anaesthesia for thyroid and parathyroid surgery: a randomised controlled trial. Anaesthesia. 2024;79(10):1072–1080. doi:10.1111/anae.16382. 39037325

[27] Beloeil H, Garot M, Lebuffe G, et al. Balanced opioid-free anesthesia with dexmedetomidine versus balanced anesthesia with remifentanil for major or intermediate noncardiac surgery. Anesthesiology. 2021;134(4):541–551. doi:10.1097/aln.0000000000003725. 33630043

[28] Chassery C, Atthar V, Marty P, et al. Opioid-free versus opioid-sparing anaesthesia in ambulatory total hip arthroplasty: a randomised controlled trial. Br J Anaesth. 2024;132(2):352–358. doi:10.1016/j.bja.2023.10.031. 38044236

[29] Wang P, Zhou X, Wang S, et al. Opioid-free anesthesia improves postoperative recovery quality of small and medium-sized surgery: a prospective, randomized controlled study. Minerva Anestesiol. 2024;90(9):759–768. doi:10.23736/s0375-9393.24.18125-4. 39279482

[30] Zhi T, Wei S, Kuang J, et al. Effects of opioid-free anesthesia combined with iliofascial nerve block on perioperative neurocognitive deficits in elderly patients undergoing hip fracture surgery: study protocol for a prospective, multicenter, parallel-group, randomized controlled trial. Trials. 2025;26(1):122. doi:10.1186/s13063-025-08828-4. 40188080 PMC11972513

[31] Chamadia S, Pedemonte JC, Hobbs LE, et al. A pharmacokinetic and pharmacodynamic study of oral dexmedetomidine. Anesthesiology. 2020;133(6):1223–1233. doi:10.1097/aln.0000000000003568. 32986820 PMC7657968

[32] Demiri M, Antunes T, Fletcher D, et al. Perioperative adverse events attributed to α2-adrenoceptor agonists in patients not at risk of cardiovascular events: systematic review and meta-analysis. Br J Anaesth. 2019;123(6):795–807. doi:10.1016/j.bja.2019.07.029. 31623842

[33] Janik A, Qiu X, Lane R, et al. Esketamine monotherapy in adults with treatment-resistant depression: a randomized clinical trial. JAMA Psychiatry. 2025;82(9):877–887. doi:10.1001/jamapsychiatry.2025.1317. 40601310 PMC12224050

[34] Rizzo A, Garçon-Poca MZ, Essmann A, et al. The dopaminergic effects of esketamine are mediated by a dual mechanism involving glutamate and opioid receptors. Mol Psychiatry. 2025;30(8):3443–3454. doi:10.1038/s41380-025-02931-3. 39972056 PMC12240861

[35] Tripodi VF, Sardo S, Ippolito M, et al. Effectiveness and safety of opioid-free anesthesia compared to opioid-based anesthesia: a systematic review and network meta-analysis. J Anesth Analg Crit Care. 2025;5(1):53. doi:10.1186/s44158-025-00272-9. 40804649 PMC12351777

