# Supplementary material for: Comparison of opioid-free versus opioid-based total intravenous anaesthesia in elderly patients undergoing short-duration surgery: a randomized controlled trial
Source: Ann Med. 2026 Feb 8;58(1):2600751. doi: 10.1080/07853890.2025.2600751 (PMC12888356; doi:10.1080/07853890.2025.2600751)
Supplement: Supplement 1 Protocol.docx [file IANN_A_2600751_SM2065.docx]

# Opioid-Free vs Opioid-Based Anesthesia in Elderly Patients Undergoing Short-Duration Surgery: A Randomized Clinical Trial

# Study protocol

Principal investigator: Chaobo Ni, MD; Huadong Ni, MD

Organizer institutes: Department of Anesthesiology and Pain Research Center, The Affiliated Hospital of Jiaxing University

Research Institutions: The Affiliated Hospital of Jiaxing University

**Introduction**

**Background and rationale**

Postoperative adverse events are a significant concern in elderly patients, often leading to prolonged hospital stays, increased healthcare costs, and diminished quality of life. Opioid-based intravenous anesthesia (OBA), while a cornerstone of traditional anesthetic practice, is associated with a high incidence of well-documented side effects, including postoperative hypoxemia, delirium (POD), and nausea and vomiting (PONV). These opioid-related adverse events are particularly pronounced in the vulnerable elderly population.

Opioid-free intravenous anesthesia (OFA) has emerged as a promising alternative strategy designed to mitigate these risks. By employing a multimodal approach with agents such as dexmedetomidine, ketamine, and lidocaine, OFA aims to provide adequate anesthesia and analgesia while minimizing or eliminating the need for intraoperative opioids. This approach may lead to improved hemodynamic stability, reduced postoperative complications, and enhanced recovery. However, despite its growing popularity, high-quality evidence from randomized controlled trials (RCTs) comparing OFA with standard OBA, specifically in the context of elderly patients undergoing short-duration, non-major surgery, remains limited. Therefore, we designed this pragmatic randomized clinical trial to rigorously evaluate the efficacy and safety of an OFA group compared to a standard OBA group in this specific patient population.

**Objectives**

We hypothesize that in elderly patients undergoing short-duration surgery, an OFA group is superior to a standard OBA group in reducing the incidence of a composite of major opioid-related adverse events. To test this hypothesis, we have designed a single-center, single-blind, randomized controlled trial aimed at assessing the efficacy and safety of OFA in preventing these adverse events in this patient population.

**Trial design**

This study is a single-center, randomized controlled trial with two parallel arms. Eligible patients will be enrolled and randomly assigned in a 1:1 ratio to receive either OFA or standard OBA. The trial is being conducted at the Affiliated Hospital of Jiaxing University in Jiaxing, China. A flowchart illustrating the study procedures is provided in Figure 1. Both groups will receive standardized perioperative management and postoperative care. Participant enrollment commenced on May 20, 2025, and was completed on December 31 2025. This work was supported by the principal investigator, Huadong Ni. The protocol reporting follows the Standard Protocol Items: Recommendations for Interventional Trials (SPIRIT) 2013 statement.

**Methods: Participants, interventions and outcomes**

**Trial setting**

The study was conducted at the Affiliated Hospital of Jiaxing University in Jiaxing, China. Written informed consent was obtained from all participants prior to enrollment.

**Eligibility criteria**

Inclusion Criteria

Participants were required to meet all of the following criteria to be eligible for enrollment:

1.Aged 60 years or older;

2.Scheduled for elective, non-major surgery with an anticipated duration of less than 90 minutes (eg, urologic or vascular procedures);

3.American Society of Anesthesiologists (ASA) physical status I to III;

4.Planned for general anesthesia with a laryngeal mask airway;

5.Able and willing to provide written informed consent.

Exclusion Criteria

Participants were excluded if they met any of the following criteria:

1.Contraindications to laryngeal mask airway use or a predicted difficult airway;

2.Severe cardiovascular disease (eg, unstable angina, New York Heart Association class III-IV heart failure);

3.Severe hepatic dysfunction (Child-Pugh class C) or renal dysfunction (estimated glomerular filtration rate <30 mL/min/1.73m²);

4.Uncontrolled diabetes (HbA1c >9%);

5.Significant pre-existing cognitive impairment (Mini-Mental State Examination [MMSE] score <18) or a history of major psychiatric disorders;

6.Chronic opioid use (defined as daily or near-daily use for >3 months) or opioid dependency;

7.Known allergy or contraindication to any of the study medications;

8.Participation in another interventional clinical trial within the past 30 days;

**Who will take informed consent?**

On the day before surgery, a member of the research team will assess eligibility criteria and provide a detailed explanation of the study to potential participants. Written informed consent will be obtained from each participant after this explanation. Participants will be afforded ample time to deliberate upon their decision and voluntarily elect to participate

**Additional consent provisions for collection and use of participant data and biological specimens**

Not applicable

**Interventions**

**Explanation for the choice of comparators**

OBA has long been the standard of care for general anesthesia, providing effective analgesia and hemodynamic control. However, its use is frequently associated with a range of adverse events, particularly in the elderly population, including postoperative hypoxemia, delirium, and nausea and vomiting. These complications underscore the clinical need for alternative anesthetic strategies that can provide comparable efficacy with an improved safety profile.

OFA has emerged as a promising alternative. By utilizing a multimodal combination of non-opioid agents, OFA is designed to minimize opioid-related adverse events while maintaining adequate anesthesia. Despite its theoretical advantages and growing adoption, there is a notable gap in high-quality evidence from randomized controlled trials directly comparing a comprehensive OFA group with standard OBA in elderly patients undergoing short-duration surgery.

Therefore, this study employs a standard, widely used OBA group as the active comparator to evaluate the clinical efficacy and safety of a multimodal OFA group. This direct comparison is intended to provide pragmatic, clinically relevant evidence to guide anesthetic choices in this vulnerable patient population and to determine if the theoretical benefits of OFA translate into a tangible reduction in postoperative adverse events.

**Intervention description**

Upon arrival in the operating room, all participants received standard monitoring, including 3-lead electrocardiography, pulse oximetry, non-invasive blood pressure, and capnography. Analgesic depth was monitored using the Surgical Pleth Index (SPI). Anesthesia was administered by the unblinded attending anesthesiologist according to the randomized allocation. In the OFA group, induction was performed with intravenous lidocaine (1 mg·kg⁻¹), esketamine (0.2-0.4 mg·kg⁻¹), and propofol (1.5-2.0 mg·kg⁻¹); maintenance was achieved with continuous infusions of propofol, esketamine (0.5 mg·kg⁻¹·h⁻¹), and dexmedetomidine (0.3-1.0 μg·kg⁻¹·h⁻¹). In the OBA group, induction was performed with intravenous sufentanil (0.2-0.4 μg·kg⁻¹) and propofol (1.5-2.0 mg·kg⁻¹); maintenance was achieved with continuous infusions of propofol and remifentanil (0.1-0.2 μg·kg⁻¹·min⁻¹). For both groups, rocuronium was used for laryngeal mask airway (LMA) insertion. The propofol infusion was titrated based on clinical signs, while the primary analgesic infusions (dexmedetomidine or remifentanil) were titrated to maintain an SPI value between 20 and 50. At the end of surgery, residual neuromuscular blockade was antagonized with intravenous sugammadex, with the dose determined by train-of-four (TOF) monitoring. The LMA was removed in the operating room once standard criteria for emergence were met. Upon arrival at the post-anesthesia care unit (PACU), all patients routinely received intravenous ketorolac 30 mg for analgesia and ondansetron 4 mg for prophylaxis against PONV. Patients were subsequently transferred from the PACU to the ward once they met standard discharge criteria.

**Criteria for discontinuing or modifying allocated interventions**

In accordance with the principles of the Declaration of Helsinki, participants have the right to withdraw from the study at any time and for any reason without prejudice to their subsequent medical care. A participant who voluntarily withdraws or is withdrawn by the investigator will be considered a dropout.

The allocated intervention may be discontinued or modified at the discretion of the attending anesthesiologist for safety reasons. Specific criteria for discontinuation include, but are not limited to:

1.Development of a severe, unexpected adverse event deemed related to the study intervention (e.g., severe hemodynamic instability unresponsive to standard treatment, anaphylaxis)；

2.The participant or their legal representative withdrawing consent during the procedure；

3.The clinical need for an open surgical conversion or a significant, unexpected prolongation of the surgery that renders the study protocol inappropriate.

Modification of the intervention, such as adjusting infusion rates beyond the pre-specified ranges, may be permitted if deemed clinically necessary by the anesthesiologist to ensure patient safety. All instances of protocol deviation, modification, or discontinuation, along with the reasons, will be meticulously documented in the case report form. All data collected up to the point of withdrawal will be included in the final analysis where possible, adhering to the intention-to-treat principle.

**Strategies to improve adherence to interventions**

To promote better adherence to the study protocol, participants will receive comprehensive information regarding the study procedures and will be thoroughly informed of potential benefits and risks. All experimental procedures will be carried out in strict accordance with the established study protocol, thereby maintaining consistency and ensuring the reliability and validity of the study results. If any concerns or health-related issues arise during the participants' involvement in the study, the research team will promptly address them.

**Relevant concomitant care permitted or prohibited during the trial**

Administration of anxiety and insomnia psychotropic medications to patients prior to surgery is strictly prohibited.

**Provisions for post-trial care**

After the completion of surgery, all patients will be transferred to a general ward, except for those in critical condition who may require admission to the Intensive Care Unit post-surgery. If any complications occur during the intervention, trial participant will receive standard postoperative care from a multidisciplinary team comprising surgical, anaesthesiology, and critical care specialists. If any serious adverse events are deemed related to this clinical trial, the research team will provide the necessary treatment free of charge. Furthermore, we will closely monitor participant throughout the postoperative period to promptly detect any potential complications and offer timely interventions as needed. These follow-up visits will not only help monitor adverse events but also provide ongoing support and encouragement, answer questions, and assist participants in addressing any issues that may emerge.

**Outcomes**

**Primary Outcome**

The primary outcome for this trial is a composite binary endpoint, defined as the occurrence of one or more of the following major opioid-related adverse events within the first 48 hours after surgery:

1.Postoperative Hypoxemia: This was defined as a pulse oximetry saturation (SpO₂) reading of less than 95% while the patient was breathing room air, lasting for at least one minute, and requiring the administration of supplemental oxygen to correct. SpO₂ was monitored continuously in the PACU and intermittently on the surgical ward as per standard institutional protocol.

2.POD: The presence of POD was assessed using the 3-Minute Disorientation Assessment Method (3D-CAM). Assessments were performed by a trained, blinded research coordinator twice daily (morning and evening) for the first two postoperative days. The 3D-CAM is a validated instrument based on the four core features of the Confusion Assessment Method (CAM): (1) acute onset and fluctuating course, (2) inattention, (3) disorganized thinking, and (4) altered level of consciousness. A diagnosis of POD was considered positive if the patient met the criteria for both feature 1 and feature 2, plus either feature 3 or feature 4.

3.PONV: This was defined as any documented episode of nausea (a subjective feeling of the urge to vomit), retching (spasmodic respiratory movements against a closed glottis), or active vomiting within the 48-hour postoperative period. The occurrence of PONV was systematically assessed by blinded nursing staff and research coordinators during routine postoperative checks and in response to patient complaints.

The occurrence of any one of these three components within the 48-hour window was sufficient to classify the patient as having met the primary composite outcome.

**Secondary and Safety Outcome**

Intraoperative Hemodynamic Stability:

1.Area Under the Curve (AUC) for MAP: Calculated as the integral of the mean arterial pressure (MAP) difference from baseline over the first 15 minutes following anesthesia induction. A more negative value indicates a greater degree of hypotension.

2.Requirement for Intraoperative Vasopressors: A binary outcome defined as the administration of at least one bolus of a vasopressor (e.g., phenylephrine or ephedrine) to treat hypotension during surgery.

Postoperative Recovery Characteristics:

1.Time to Extubation: Measured in minutes, from the cessation of all anesthetic infusions to the removal of the LMA.

2.Length of Stay in PACU: Measured in minutes, from arrival in the PACU to meeting standard discharge criteria.

3.Postoperative Pain Scores: Assessed using the Numerical Rating Scale (NRS; 0-10 scale, where 0 is no pain and 10 is the worst imaginable pain) at rest and during movement at 24 and 48 hours postoperatively.

3.Severity of PONV: Assessed using a 4-point ordinal scale (0=none, 1=mild, 2=moderate, 3=severe) at 24 and 48 hours postoperatively.

Safety Outcomes

Safety was assessed by recording the incidence of the following adverse events:

1.Injection Pain: Patient-reported pain at the intravenous site during the administration of induction agents, assessed immediately after induction.

2.Bradyarrhythmia: Defined as any episode of sinus bradycardia (heart rate < 50 beats/min) or atrioventricular block requiring intervention, documented intraoperatively.

3.Tachycardia: Defined as a sustained sinus tachycardia (heart rate > 100 beats/min) not attributable to surgical stimulation, documented intraoperatively.

4.Headache and Dizziness: Patient-reported symptoms documented during the PACU stay and up to 48 hours postoperatively.

5.Emergence Agitation: Clinically assessed by the PACU nursing staff as any episode of agitation or restlessness during recovery from anesthesia requiring intervention.

**Participant timeline**

The schedule of enrollment, interventions, and assessments is shown in eTable. 1

**Sample size**

The sample size for this trial was determined based on the primary composite outcome: the incidence of one or more major opioid-related adverse events within 48 hours postoperatively.The calculation was informed by a review of published literature and institutional audit data. We estimated the baseline incidence of the primary composite outcome in the standard OBA group to be approximately 40%. We hypothesized that the OFA group would be superior and targeted a clinically meaningful relative risk reduction of 40%, corresponding to an expected incidence of 24% in the OFA group.

To detect this difference with a statistical power of 80% at a two-sided significance level (α) of 0.05, a minimum of 130 patients per group was required. To account for a potential dropout rate of approximately 15%, we inflated the sample size. Therefore, the final target sample size was set at 400 participants in total (200 participants per group). This sample size was also deemed adequate for the subgroup analyses. The power calculation was performed using PASS software (version 15.0, NCSS, LLC).

**Recruitment**

Patients will be recruited at Affiliated Hospital of Jiaxing University, China.

**Assignment of interventions: allocation**

**Sequence generation**

The randomization will be conducted using SPSS software version 26.0, which will generate a random number table maintaining a 1:1 allocation ratio between the OFA group and OBA group. The randomization will utilize a block size of 4 or 6 and will be performed by a statistician who is not directly involved in the study. As a key component of the study design, randomization aims to ensure the fairness of the allocation process and to reduce the impact of potential confounding variables. Employing computerized randomization supervised by a statistician enhances the scientific rigor and integrity of our study.

**Concealment mechanism**

To ensure the integrity of the randomization process, the randomization plan and group allocations will be securely sealed in closed, opaque envelopes. Furthermore, the random sequence will be concealed from the researchers by employing blinding procedures. This approach aims to reduce the risk of selection bias and ensure that participants are fairly assigned to their respective study groups.

**Implementation**

On the day of surgery, after an eligible participant provided written informed consent, the randomization process was implemented. An independent research assistant, not involved in patient assessment or care, selected the next sequentially numbered, sealed, opaque envelope corresponding to the appropriate stratum (type of surgery: urologic vs. non-urologic).

The envelope was opened in a private area by the attending anesthesiologist, who was responsible for preparing the anesthetic drugs. The card inside revealed the treatment allocation (OFA or OBA) for that participant. This process ensured that the allocation was concealed from the patient and all other study personnel, including outcome assessors and data analysts, until the moment of intervention. Participants were informed at the time of consenting that they had an equal chance of being assigned to either group.

**Assignment of interventions: Blinding**

**Who will be blinded**

This was a single-blind trial designed to maintain blinding for all personnel where feasible. Participants were blinded to their treatment allocation throughout the study. The research staff responsible for postoperative outcome assessment, as well as the data analysts and principal investigators, were also strictly blinded to group assignments. The attending anesthesiologists could not be blinded due to the distinct nature of the anesthetic ways; however, they were not involved in any postoperative data collection or assessment to minimize potential bias.

**Procedure for unblinding if needed**

Unblinding is permissible if necessary for safety reasons.

**Data collection and management**

**Plans for assessment and collection of outcomes**

Data for this study were collected prospectively using a standardized electronic case report form (eCRF) and the electronic medical record system. All research personnel involved in data collection underwent formal training on the study protocol and assessment tools prior to the trial. Data collection was structured across preoperative, intraoperative, and postoperative phases. Preoperative data, including baseline demographics and medical history, were collected by a blinded research coordinator. Intraoperative data, such as drug dosages and surgical details, were recorded by the unblinded attending anesthesiologist. All postoperative outcomes were collected by trained research coordinators and nursing staff who were strictly blinded to treatment allocation. The primary composite outcome and its components were assessed at standardized intervals up to 48 hours postoperatively. Secondary and safety outcomes were recorded as they occurred or at pre-specified time points. To ensure data quality, regular checks were performed, and all collected information was treated as confidential and stored securely.

**Plans to promote participant retention and complete follow-up**

In this clinical trial, all participants will undergo a comprehensive 48h postoperative follow-up. Prior to obtaining informed consent, participants will be provided with detailed information about the study protocol and procedures to ensure thorough understanding and active cooperation. Our research team is dedicated to addressing any unforeseen issues that may arise during the follow-up period, including patient discomfort or complications, with the aim of facilitating thorough and precise postoperative assessments.

**Data management**

All participant data will be meticulously recorded in the eCRFs. Subsequently, these data will be entered into Microsoft Excel by an investigator, with thorough verification through double-checking. Raw data, including eCRFs, will be securely stored in a locked cabinet within the anesthesiology office. Electronic data will be housed on a computer equipped with dual password protection. Access to these files will be restricted to authorized personnel only.

**Confidentiality**

All researchers are committed to complying with regulations regarding patient confidentiality and ethical standards. Personal data recorded during the study are handled with the highest level of confidentiality and managed in accordance with data protection laws. Confidential information is securely processed and stored to ensure compliance with privacy regulations.

**Plans for collection, laboratory evaluation and storage of biological specimens for genetic or molecular analysis in this trial/future use**

Not applicable

**Statistical methods**

**Statistical methods for primary and secondary outcomes**

All statistical analyses were conducted according to the intention-to-treat (ITT) principle, which included all randomized participants. Statistical analyses were performed using SAS software (version 9.4 or later). A two-sided P value < .05 was considered statistically significant for all analyses.

The primary outcome, a composite binary endpoint, was analyzed using a multivariable logistic regression model to estimate the adjusted odds ratio (aOR) and its 95% CI for the OFA group compared with the OBA group. The model was adjusted for pre-specified baseline covariates, including age, sex, body mass index (BMI), ASA physical status, Charlson Comorbidity Index, and educational level. To account for potential nonlinearity, age and BMI were modeled using restricted cubic splines. The number needed to treat (NNT) was calculated from the adjusted risk difference derived from a supplementary modified Poisson regression model. Missing data for the primary outcome were handled using multiple imputation.

For secondary outcomes, the area under the curve for mean arterial pressure (MAP AUC) was compared between groups using an analysis of covariance (ANCOVA) model, with the baseline MAP value as a covariate. Other continuous secondary outcomes were compared using the independent samples t-test or the Mann-Whitney U test, as appropriate based on data distribution. Ordinal outcomes, such as the severity of PONV, were analyzed with the Mann-Whitney U test. Binary secondary and safety outcomes, including the individual components of the primary endpoint, were compared using the χ² test or Fisher's exact test.

**Interim analyses**

There is no interim analysis.

**Methods for additional analyses (e.g. subgroup analyses)**

A series of additional analyses were conducted to assess the consistency of the primary finding and to generate further hypotheses. Subgroup analyses for the primary outcome were performed based on type of surgery (urological vs non-urological), age (<75 vs ≥75 years), and Charlson Comorbidity Index (<5 vs ≥5). The consistency of the treatment effect across subgroups was evaluated by testing for a statistical interaction between the treatment assignment and the subgrouping variable in the logistic regression model. To assess the robustness of the primary finding, we conducted several sensitivity analyses, including a per-protocol analysis and a comparison between the primary multivariable model and an unadjusted model. Additionally, two exploratory analyses were performed: a series of univariable logistic regression analyses to identify potential predictors of the primary outcome, and a restricted cubic spline analysis to examine the dose-response relationship between cumulative propofol dose and time to extubation.

**Methods in analysis to handle protocol non-adherence and any statistical methods to handle missing data**

Given our comprehensive pre-anesthesia evaluation and detailed explanation of the intervention, along with a strong emphasis on patient cooperation during the informed consent process, we anticipate minimal protocol non-adherence among patients. In the event of missing data, multiple imputation will be employed for analysis, supplemented by sensitivity analysis.

**Plans to give access to the full protocol, participant level-data and statistical code**

The data and protocols of this trial are available only to the trial leader, and there is no right to access the data of the participants in this trial without first obtaining permission from the principal leader

**Oversight and monitoring**

**Composition of the coordinating centre and trial steering committee**

Not applicable

**Composition of the data monitoring committee, its role and reporting structure**

A formal data monitoring committee was not established for this trial. Given that the study interventions consisted of anesthetic drugs and techniques in routine clinical use, the overall risk to participants was considered low. Furthermore, the trial was of a relatively short duration and moderate sample size. Ongoing safety monitoring was conducted by the principal investigator and the local institutional review board (IRB) as part of their standard oversight responsibilities.

**Adverse event reporting and harms**

All adverse events (AEs) and serious adverse events (SAEs) were systematically monitored and documented from the time of randomization until 48 hours postoperatively. An AE was defined as any untoward medical occurrence in a participant, regardless of whether it was considered related to the study intervention. An SAE was defined as any AE that resulted in death, was life-threatening, required inpatient hospitalization or prolongation of existing hospitalization, or resulted in persistent or significant disability.

The unblinded attending anesthesiologist was responsible for identifying and documenting any intraoperative AEs. Blinded research coordinators and nursing staff were responsible for identifying and documenting postoperative AEs through systematic assessment and patient interviews.

All AEs were recorded on a dedicated page in the electronic case report form, detailing the event, its onset and resolution, severity (mild, moderate, or severe), and the investigator's assessment of its relationship to the study intervention (definitely related, probably related, possibly related, or not related). All SAEs were required to be reported to the principal investigator within 24 hours of awareness and subsequently to the IRB in accordance with local regulatory requirements and institutional policies.

**Frequency and plans for auditing trial conduct**

A researcher not involved in the trial was selected as an independent reviewer, who reviewed the trial process, data collection, and assessment of form-level informed consent every two weeks.

**Plans for communicating important protocol amendments to relevant parties (e.g. trial participants, ethical committees)**

The trial was reviewed and approved by the Ethics Committee of The Affiliated Hospital of Jiaxing University according to this protocol. In principle, the protocol cannot be modified unless the ethical committee agrees.

**Dissemination plans**

Upon completion of the statistical analysis, we will make every effort to publish the results in reputable peer-reviewed journals specializing in clinical anesthesia.

**Discussion**

This single-center, single-blind, randomized controlled trial is designed to rigorously evaluate whether an OFA group is superior to a standard OBA group in reducing a composite of clinically significant adverse events in elderly patients undergoing short-duration surgery. To our knowledge, while the concept of OFA is gaining traction, high-quality evidence from a pragmatic trial focusing on this specific, vulnerable patient population and surgical context remains scarce. This study aims to address this critical evidence gap.

The choice of our primary outcome—a composite of postoperative hypoxemia, delirium, and PONV—is a key strength of this trial. These are not only common and burdensome complications in the elderly but are also strongly linked mechanistically to the use of opioids. By combining them into a single endpoint, we increase the statistical power to detect a clinically meaningful overall benefit of the OFA strategy. Furthermore, our comprehensive panel of secondary and safety outcomes, particularly the objective measures of hemodynamic stability (MAP AUC and vasopressor use), will provide a nuanced understanding of the broader physiological impact of the two anesthetic approaches.

Our study design incorporates several methodological strengths to ensure the validity and reliability of the findings. The use of centralized, stratified randomization and strict blinding of patients, outcome assessors, and data analysts minimizes the risk of selection and detection bias. The detailed, standardized anesthetic protocol, including the use of the SPI to guide analgesic titration, enhances the reproducibility of our intervention. Moreover, our robust statistical analysis plan, which includes pre-specified subgroup and sensitivity analyses and modern techniques like restricted cubic splines and multiple imputation, is designed to yield reliable and nuanced conclusions.

We acknowledge several potential limitations. First, as a single-center study, the generalizability of our findings to other institutions with different patient populations or clinical practices may be limited. Second, the attending anesthesiologists could not be blinded to the treatment allocation, which could introduce a risk of performance bias. We have sought to mitigate this by standardizing the protocol as much as possible and by ensuring that all outcome assessors are strictly blinded. Third, while our composite endpoint is clinically relevant, the overall benefit could be driven by a large effect on one component (e.g., PONV) and a smaller or null effect on others. The analysis of individual components will help clarify this.

In conclusion, this randomized controlled trial is poised to provide high-quality, clinically relevant evidence on the efficacy and safety of an OFA group in a common and vulnerable surgical population. If our hypothesis is confirmed, the findings could significantly influence clinical practice by providing a strong rationale for adopting OFA to improve perioperative outcomes and enhance patient safety in the elderly.

**eTable 1. Study Timeline**

| **Timepoint** | **Enrolment** (≤14 d) | **Allocation** (Day 0, pre-induction) | **During Surgery** | **End of surgery** | **PACU** (0–2 h) | **The first day after the surgery** | **The second day after the surgery** | **Discharge** |
| --- | --- | --- | --- | --- | --- | --- | --- | --- |
| **Eligibility screen** | X |  |  |  |  |  |  |  |
| **Informed consent** | X |  |  |  |  |  |  |  |
| **Baseline demographics, comorbidities, meds** | X |  |  |  |  |  |  |  |
| **Randomization (1:1, stratified)** |  | X |  |  |  |  |  |  |
| **Intervention: OFA-TIVA / OBA-TIVA** |  |  | X |  |  |  |  |  |
| **Baseline MAP (pre-induction)** |  | X |  |  |  |  |  |  |
| **Hemodynamics: MAP AUC (0–15 min)** |  |  | X |  |  |  |  |  |
| **Vasopressor use** |  |  | X |  |  |  |  |  |
| **Anesthetic drugs** |  |  | X |  |  |  |  |  |
| **Extubation time** |  |  |  | X |  |  |  |  |
| **PACU length of stay** |  |  |  |  | X |  |  |  |
| **Pain (NRS) at rest / movement** |  |  |  |  | X | X |  |  |
| **PONV severity (0–3)** |  |  |  |  | X | X | X |  |
| **Hypoxemia episodes** |  |  |  |  | X | X | X |  |
| **POD (3D-CAM)** |  |  |  |  |  | X | X |  |
| **Safety AEs: injection pain** |  |  | X |  |  |  |  |  |
| **Safety AEs: bradyarrhythmia / tachycardia** |  |  | X |  |  |  |  |  |
| **Safety AEs: headache / dizziness** |  |  |  |  | X |  |  |  |
| **Emergence agitation** |  |  |  | X | X |  |  |  |
| **Concomitant medications** | X |  | X |  |  |  |  |  |
| **Protocol deviations / withdrawals** |  |  | X | X | X | X | X | X |
| **Adverse event reporting** |  |  | X | X | X | X | X | X |
| **Data review / lock** |  |  |  |  |  |  |  | X |
