# Supplementary figures and images for: Comparison of opioid-free versus opioid-based total intravenous anaesthesia in elderly patients undergoing short-duration surgery: a randomized controlled trial
Source: Ann Med. 2026 Feb 8;58(1):2600751. doi: 10.1080/07853890.2025.2600751 (PMC12888356; doi:10.1080/07853890.2025.2600751)

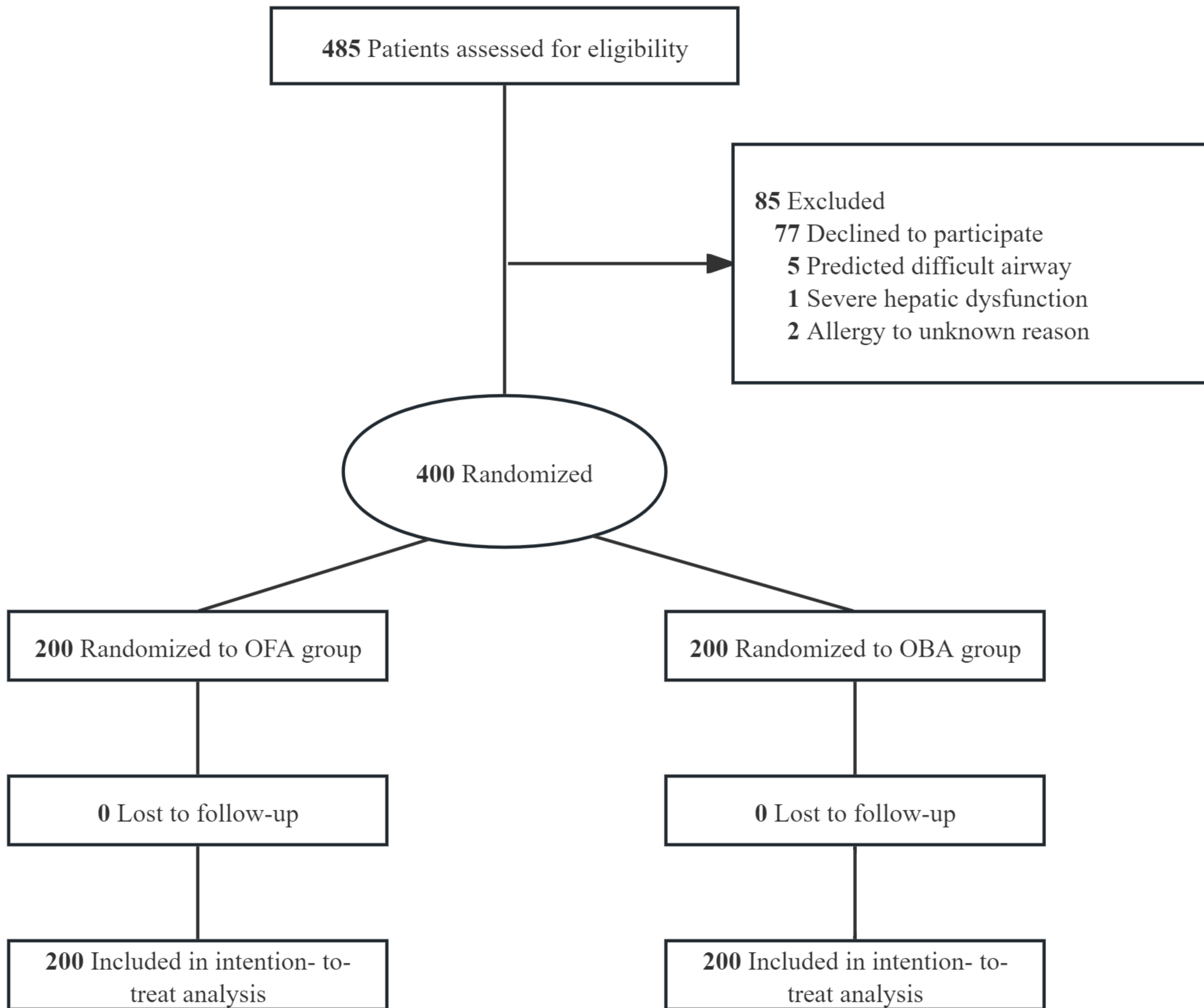

Supplement: consort flow chart.pdf [file IANN_A_2600751_SM2064.pdf]
